# Focus on Autoimmune Myocarditis in Graves' Disease: A Case-Based Review

**DOI:** 10.3389/fcvm.2021.678645

**Published:** 2021-07-07

**Authors:** Lujin Wu, Wei Wang, Qianru Leng, Nana Tang, Ning Zhou, Yan Wang, Dao Wen Wang

**Affiliations:** ^1^Division of Cardiology, Department of Internal Medicine, Tongji Medical College, Tongji Hospital, Huazhong University of Science and Technology, Wuhan, China; ^2^Hubei Key Laboratory of Genetics and Molecular Mechanism of Cardiologic Disorders, Huazhong University of Science and Technology, Wuhan, China; ^3^Nursing Teaching Office of Internal Medicine, Tongji Hospital Affiliated to Tongji Medical College of Huazhong University of Science & Technology, Wuhan, China

**Keywords:** Graves' disease, thyrotoxicosis, autoimmune myocarditis, cMRI, glucocorticoid

## Abstract

The manifestations of hyperthyroidism-related myocardial damage are multitudinous, including arrhythmia, dilated cardiomyopathy, valvular diseases, and even cardiogenic shock. Acute myocarditis induced by thyrotoxicosis had been reported in a few studies. However, attention on its prevalence and underlying mechanisms is sorely lacking. Its long-term harm is often ignored, and it may eventually develop into dilated cardiomyopathy and heart failure. We report a case of Graves' disease with a progressive elevation of hypersensitive cardiac troponin-I at several days after discontinuation of the patient's anti-thyroid drugs. Cardiac magnetic resonance imaging (CMRI) showed inflammatory edema of some cardiomyocytes (stranded enhanced signals under T2 mapping), myocardial necrosis (scattered enhanced signals under T1 late gadolinium enhancement) in the medial and inferior epicardial wall, with a decreased left ventricular systolic function (48%), which implied a possibility of acute myocarditis induced by thyrotoxicosis. The patient was then given a transient glucocorticoid (GC) treatment and achieved a good curative effect. Inspired by this case, we aim to systematically elaborate the pathogenesis, diagnosis, and treatment of hyperthyroidism-induced autoimmune myocarditis. Additionally, we emphasize the importance of CMRI and GC therapy in the diagnosis and treatment of hyperthyroidism-related myocarditis.

## Introduction

Cardiovascular disease remains one of the largest causes of death worldwide. Thyroid hormones are closely related to the cardiovascular system (CVS) in both physiological and pathological situations. Dating back to ontogeny, the thyroid and the CVS are derived from the same embryological origin. The former modulates each component of the latter for a normal function during the developmental stage (1). Thyroid hormone dysfunction has been shown to be devastating to the heart, and both all-cause and cardiovascular mortality are increased in hyperthyroidism (2).

Classic hyperthyroidism cardiomyopathy was defined as a range of heart diseases caused by hyperthyroidism, mainly manifesting as arrhythmia, atrial fibrillation, cardiac enlargement, heart failure, and valvular diseases (3). Its severity is second only to hyperthyroidism crisis, which is an important cause of death in hyperthyroidism patients (4). Currently, an interesting case with Graves' disease (GD) was admitted to our hospital, shifting our attention to acute myocarditis induced by hyperthyroidism. Progressive elevation of high-sensitivity cardiac troponin I (hs-cTnI) without any discomfort was her main clinical feature. The features of cardiac magnetic resonance imaging (CMRI) met the upgraded Lake Louise criteria (LLC) in 2018 for myocarditis rather than coronary ischemia.

Myocarditis is broadly defined as an inflammatory process invading cardiomyocytes (5). Viral myocarditis is the most frequent type, often with fever or other symptoms of infection, chest pain, dyspnea, electrocardiogram (ECG) changes, and troponin elevation (6). There is strong evidence that autoimmune diseases are also involved in the occurrence of acute myocarditis (7). Furthermore, 7.2% of myocarditis patients and 15% of fulminant myocarditis had autoimmune diseases (8). Although GD is a common autoimmune thyrotoxicosis and well-known to be related to chronic heart failure (especially dilated cardiomyopathy), hyperthyroidism-associated acute myocarditis is rarely reported. However, retrospective studies reported that 9–16% of unexplained non-ischemic dilated cardiomyopathy cases have a histological evidence of myocarditis (9). Thus, there may exist huge omissions about hyperthyroidism-related myocarditis in GD patients.

Additionally, our patient showed a good response to transient anti-inflammatory therapy with glucocorticoid (GC). Based on the treatment experience from this patient, we will systematically elaborate the following scientific issues: (1) the potential mechanisms and characteristics of thyrotoxic-related myocarditis, (2) the diagnosis and antidiastole about thyrotoxic-related myocarditis, (3) the value of CMRI in the diagnosis of inflammatory cardiomyopathy, and (4) potential treatment strategies of thyrotoxic-related myocarditis.

## Case Presentation

A 31-year-old woman with 2-month pregnancy was diagnosed with hyperthyroidism 3 years ago and mainly complained with palpitation and excessive sweating. Then, she accepted propylthiouracil treatment orally and persisted 6 months after her delivery. Her above-mentioned symptoms were significantly improved, and she gained healthy birth outcomes. The doctors later switched her medication to methimazole (5 mg, b.i.d.). Both drugs showed good control for her disease without any adverse events. On July 10, 2020, she voluntarily discontinued the medications without any medical consultation. At 10 days later (July 20), she was re-admitted at our hospital for further treatment because her thyroid function examination showed obvious abnormalities without any discomfort. Except for laparoscopic surgery for ovarian cyst in 2015, she denied any other history of surgery, chronic diseases, inherited diseases, and allergy.

**Figure 1:**
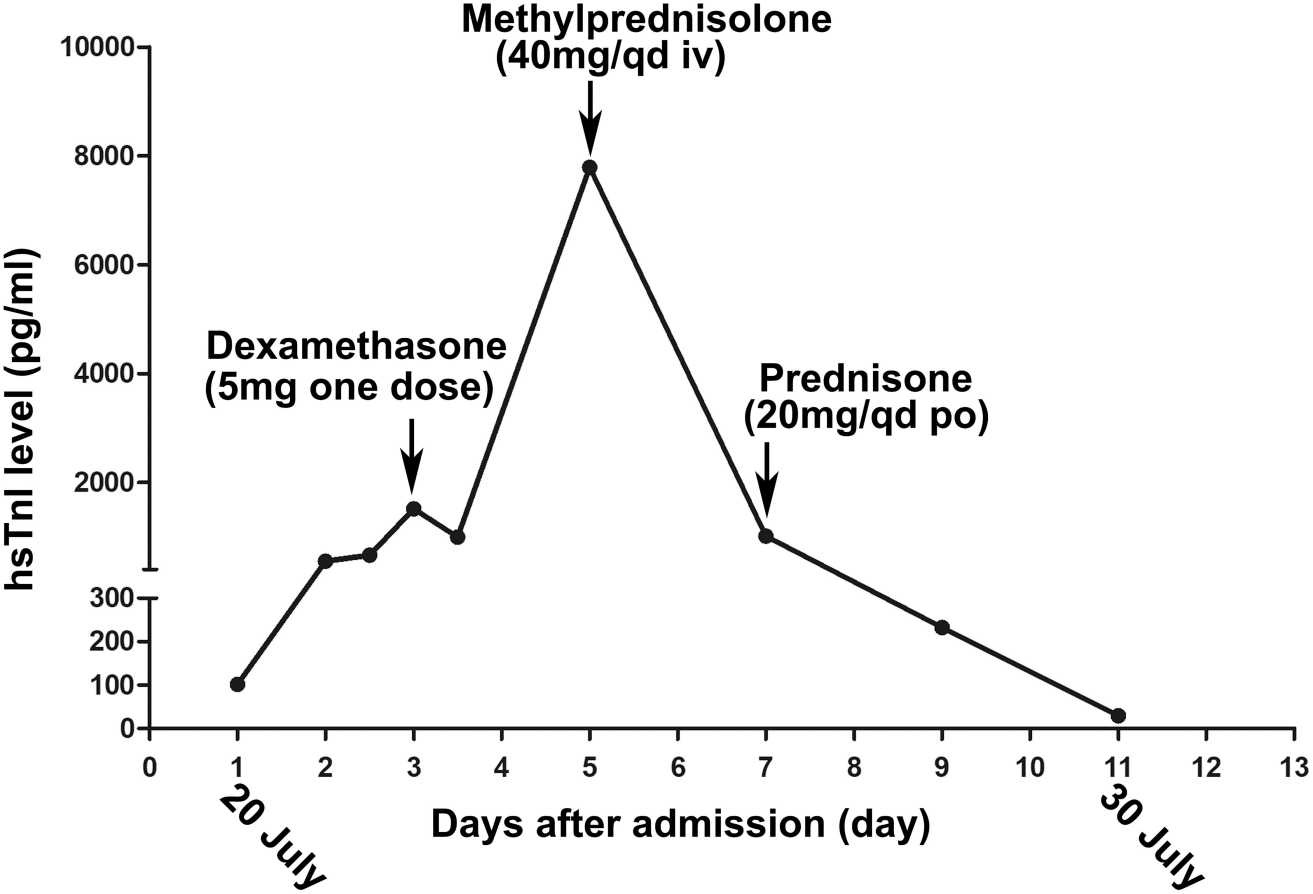
The changing trend of the high-sensitivity cardiac troponin I (hs-cTnI) during her hospitalization. The arrow indicates the times when the patient accepts glucocorticoid treatment.

**Figure 2:**
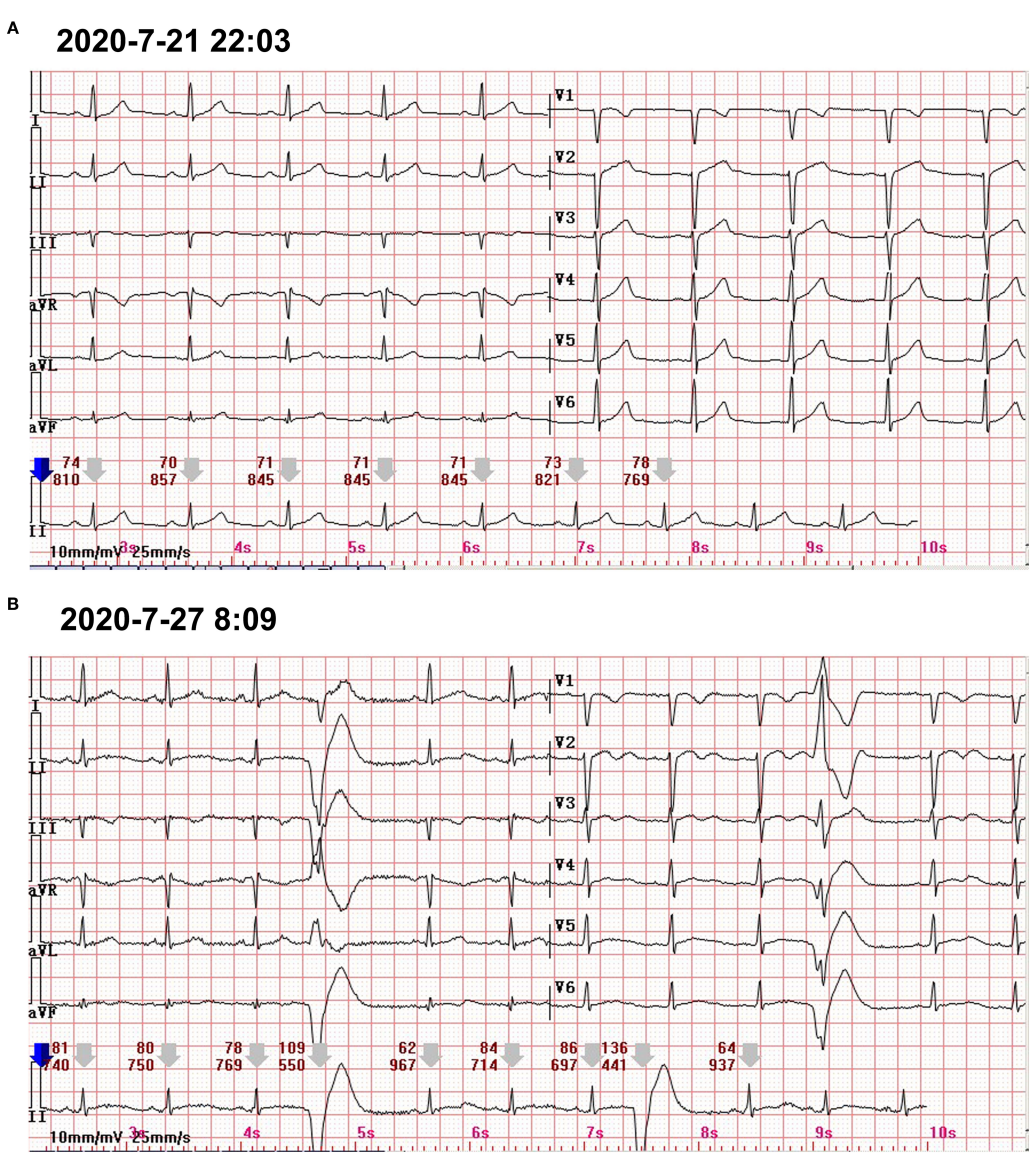
**(A)** The 12-lead standard ECG only showed the poor progression of R waves in V1–V3. **(B)** A 24-h Holter ECG captured the occasional premature ventricular contractions.

Upon admission, the physical examination showed that the heart rate was 96 beats/min and the thyroid gland was of grade II enlargement. The laboratory workup showed that the patient had decreased thyroglobulin (Tg), 0.33 μg/L, with positive thyroglobulin antibody (Tg-Ab) >4,000.00 IU/ml, positive thyroid peroxidase antibody (TPO-Ab) >600.00 IU/ml, and positive thyroid-stimulating hormone (TSH) receptor antibody (TR-Ab) 7.08 IU/L. Her thyroid function showed decreased TSH (0.005 μIU/ml) and increased free thyroxine (FT4, 51.27 ng/L) and free triiodothyronine (FT3, 16.19 pg/ml; Table 1). No obvious abnormality was found in the blood routine test, liver and kidney function, electrolyte, urine routine test, D-dimer, ESR, hsCRP (Table 2), and NT-proBNP. Surprisingly, the hs-cTnI was increased by 101.5 pg/ml (normal range, <15.6 pg/ml) (Figure 1). In addition, the 12-lead ECG showed poor progression of R waves in leads V1–V3 (Figure 2A). The echocardiography showed that the heart morphology was normal without valvular disease and segmental wall motion, and the value of ejection fraction (EF) was 70%. No abnormality was found by color Doppler ultrasound about the liver, gallbladder, spleen, pancreas, kidney, and bladder, except for multiple calculi in the intrahepatic duct.

**Table 1:** Laboratory parameters of thyroid function (FT3, FT4, and TSH).

**Date**	**FT3 (pg/L)**	**FT4 (ng/L)**	**TSH (μIU/L)**
Normal range	2.0–4.4	9.32–17.09	0.27–4.2
21 July	16.19	51.27	<0.005
25 July	12.35	50.44	<0.005
28 July	7.65	38.1	<0.005
30 July	6.89	33.79	<0.005

**Table 2:** Clinical indicators about infection.

**Respiratory symptoms**	**None**	**Anti-CMV IgM**	**Negative**
**Clinical indicators**			
Temperature (°C)	36.1 (36.1–37)	Anti-HSV-I IgM	Negative
Neutrophils (10^9^/L)	2.10 (1.80–6.30)	Anti-HSV-II IgM	Negative
Lymphocytes (10^9^/L)	1.38 (1.10–3.20)	Anti-EBV IgM	Negative
Hemoglobin (g/L)	118 (115.0–150.0)	Anti-PVB19 IgM	Negative
Platelets (10^9^/L)	223 (125.0–350.0)	Anti-CVB IgM	Negative
hsCRP (mg/L)	3.8 (<10)	Anti-CA16 IgM	Negative
ESR (mm/H)	6 (0–15)	Anti-ECHO IgM	Negative
TG (μg/L)	0.33 (3.5–77)	Anti-MV IgM	Negative
TG-Ab (IU/ml)	>4,000 (0–115)	Anti-VZV IgM	Negative
TPO-Ab (IU/ml)	>600 (0–34)	Anti-RV IgM	Negative
TRAb (IU/L)	7.08 (0–1.58)	Anti-TOX IgM	Negative

*hsCRP, high-sensitivity C-reactive protein; ESR, erythrocyte sedimentation rate; TG, thyroglobulin; TG-Ab, thyroglobulin antibody; TPO-Ab, thyroid peroxidase antibody; TRAb, thyrotropin receptor antibody; CMV, cytomegalovirus; HSV, herpes simplex virus; EBV, Epstein–Barr virus; PVB19, parvovirus B19; CVB, coxsackievirus B; CA16, coxsackievirus A16; ECHO, enteric cytopathic human orphan virus; MV, measles virus; VZV, varicella-zoster virus; RV, rubella virus; TOX, toxoplasma*.

**Figure 3:**
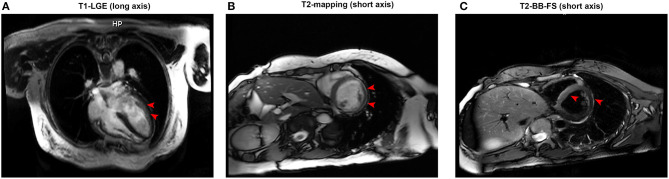
**(A)** Representative late gadolinium enhancement (red arrow) in the left ventricular anterior wall under T1 imaging (long axis). **(B)** An enhancement signal (red arrow) appeared in the inferior wall and inferolateral wall of the middle segment of the left ventricle under T2 mapping (short axis). **(C)** An enhanced signal (red arrow) appeared in the interventricular septum in T2 black blood fat suppression sequences (short axis).

At the following 2 days, the level of hs-cTnI was gradually increased to 1,516.3 pg/ml. The results of a re-examination of both ECG and echocardiography were similar to the previous results. Although the patient did not express any discomfort, the elevation of hs-cTnI and abnormal ECG suggested that myocardial necrosis existed. In addition to prophylactic antiplatelet (aspirin), statins, and anti-hyperthyroidism drug methimazole, 5 mg dexamethasone sodium phosphate (once) was tried intravenously at 10:28 on July 22. At 16:27 on the same day, the hs-cTnI was surprisingly found to drop to 994.6 pg/ml, rapidly. However, the level of hs-cTnI rose dramatically to its peak 7,785.9 pg/ml for the next 2 days (Figure 1). During this period, the only changed treatment was that the glucocorticoids were discontinued. Moreover, 24-h Holter ECG captured the occasional premature ventricular contractions (Figure 2B). Although the patient still had no abnormal symptoms about the heart, percutaneous coronary angiography (PCA) and CMRI were recommended. She rejected the invasive PCA. The CMRI showed an inflammatory edema of the myocardium [stranded enhanced signals under T2 mapping and ventricular septal enhancement signal in T2 black blood fat suppression (T2-BB-FS) sequences], myocardial necrosis [scattered enhanced signals under T1 late gadolinium enhancement (LGE)] in the medial and inferior epicardial wall, with a decreased left ventricular systolic function (LVEF, 48%), which are consistent with the change of acute myocarditis (Figure 3). Additionally, she does not appear to have been infected by any pathogens since the results of serotype antibodies for several viruses showed that the IgM of all selected viruses are negative, including CMV, HSV-I, HSV-II, EBV, PVB19, CVB, CA16, ECHO, MV, VZV, RV, and TOX. Thus, methylprednisolone (40 mg/d) was given intravenously. Considering no risk factors and evidence for coronary heart disease, antiplatelet and statins were withdrawn in the meantime. At the sixth day after admission (26 July), her hs-cTnI was significantly decreased to 1,014.8 pg/ml. Then, intravenous methylprednisolone was changed to oral prednisone (20 mg/day) for the next 4 days. The patient's myocardial injury markers gradually decreased and fell to the normal range (29.4 pg/ml) when she was discharged from our hospital on July 30.

## Discussion

Although direct evidence to exclude coronary artery occlusion in this patient is lacking, the CMRI result seemed not consistent with the characteristics of myocardial infarction. Interestingly, hs-cTnI decreased within hours after the first temporary administration with 5 mg dexamethasone but rapidly increased by 7.8-fold in the next 2 days once GC was withdrawn. When the GC treatment became continuous, the myocardial injury markers decreased steadily. These features are more in line with the characteristics of myocarditis. In addition, the facts that the patient did not have fever and respiratory symptoms and her blood routine, inflammatory markers, and serum IgM of several viruses were normal ruled out bacterial or viral infection (Table 2). Therefore, we conjectured that this patient suffered from acute autoimmune myocarditis caused by thyrotoxicosis. However, current evidence for the concept of thyrotoxicosis-induced myocarditis seem to be limited. There are only some scattered reports and few studies with small sample sizes about myocarditis caused by hyperthyroidism (10–12). Inspired by the successful experience of the patient, we believe that it is necessary and meaningful to deeply elaborate this disease and summarize the current related research. Although more clinical studies are urgently need, we try to introduce the epidemiological data, mechanism, diagnostic methods, and treatment strategies for thyrotoxicosis-induced myocarditis in this study.

### Epidemiological Characteristics

Hyperthyroid cardiomyopathy is a general term for a series of heart diseases, including arrhythmia, cardiac hypertrophy, heart failure, and valvular diseases, which are precipitated by Graves' disease (GD) (3). Thyrotoxicosis-related myocarditis can be differently defined as the inflammatory response damaging the muscular tissues of heart caused by thyrotoxicosis (13). Thyrotoxic cardiomyopathy has been reported as an initial presentation in 6% of hyperthyroidism patients (14). Compared with arrhythmia, congestive heart failure, and dilated heart disease, thyrotoxicosis-related myocarditis is relatively rare and occasionally reported in few studies (15). Mavrogeni et al. investigated 250 patients with hyperthyroidism, 50 of whom had persistent cardiac symptoms, including chest pain, dyspnea, and palpitations. Fifteen of 50 (30%) had been confirmed as inflammatory edema of the cardiomyocytes by CMRI. In addition, three patients were further confirmed by myocardial (or endomyocardial) biopsy as lymphocyte infiltration, but not any evidence of viral infection was found (10). Although accurate epidemiological information is lacking, we can speculate that the incidence rate of thyrotoxicosis-related myocarditis may be seriously underestimated. On the one hand, many cases of myocarditis are likely underdiagnosed due to subclinical or non-specific symptoms; on the other hand, CMRI or myocardial biopsy, which has an important value in the diagnosis of myocarditis, is not widely used in the clinic due to their limitations. In 2000, WD Edwards and his colleagues analyzed 11 biopsied patients with GD with reduced ejection fraction. The results showed that 18.2% (2/11) of the patients presented with lymphocytic myocarditis, and the other important manifestations were dilated cardiomyopathy (6/11) and arrhythmogenic right ventricular dysplasia (3/11) (12). In addition, a recent study which analyzed 173 patients with myocarditis, as confirmed by cardiac biopsy, found that about 23/173 (13.3%) of them had hyperthyroidism, and the main cause was autoimmune myocarditis (19/23 = 82.6%) (16). These results suggest that thyrotoxicosis-related myocarditis needs be paid more attention.

### Underlying Mechanism

Myocarditis is an inflammatory disease of the myocardium and associated with immune dysfunction which may frequently lead to the development of dilated cardiomyopathy (17). Three distinct forms of myocarditis are recognized: idiopathic, autoimmune, and infectious. Some autoimmune and auto-inflammatory diseases, such as sarcoidosis, Behçet's disease, eosinophilic granulomatosis, myositis, and systemic lupus erythematosus, had been well-documented to cause myocarditis in previous researches (18, 19). In recent years, some studies found that Graves' disease, another autoimmune disease, can also develop into inflammatory cardiomyopathy (8, 20). In 1999, Yagoro et al. firstly identified lymphocyte infiltration by endomyocardial biopsy in a patient with autoimmune thyroiditis and confirmed the presence of anti-cardiac antibodies in the plasma or myocardium (21). Later, some studies had confirmed its pathological change as lymphocyte-dominated autoimmune myocarditis (12, 22). Due to the lack of animal models of myocarditis caused by thyrotoxicosis, the mechanism of myocarditis is poorly understood. What is clear, however, is that the crosstalk between cardiomyocyte injury and inflammation dysfunction should be the key steps.

#### The Pathophysiological Mechanism of Myocardial Damage

The damage of hyperthyroidism on the cardiovascular system had long been well-recognized (23), involving hyperdynamic, hypermetabolism, genomic, and non-genomic molecular regulation. Clinical findings indicate that excessive elevated thyroid hormone increased the heart rate and myocardial contractility and relaxed the peripheral vessels, leading to a significant increase in cardiac output. However, a long period of hyperdynamic state is not good for the heart (3), which was also described as thyrotoxicosis. Firstly, a significant increase of cardiac load can eventually lead to compensatory cardiac hypertrophy and gradually develop into heart failure. Secondly, increased myocardial metabolism and oxygen consumption lead to mitochondrial dysfunction and oxidative stress injury (24). Thirdly, heart relative ischemia aggravates cardiomyocyte injury for shortening of the coronary artery filling time. Myocardial injury can trigger inflammatory responses that have been demonstrated in several basic studies, such as in transverse aortic constriction mice or myocardial infarction mice (25, 26).

Additionally, thyroid hormone also changes myocardial function by regulating gene expression and ion channel state, which provides a molecular biological perspective for us to understand the myocardial damage of hyperthyroidism (27). Both thyroid hormone (TH) receptors and two T3-binding nuclear receptors, TRa1 and TRb1, are expressed in the cardiac myocyte (2). The latter mediates the binding of TH to TH response elements in the promoter regions of TH-responsive genes. Those genes encoding important structural and regulatory proteins, including myosin heavy chain isoforms α and β, sarcoplasmic reticulum calcium-activated ATPase (SERCA2), phospholamban, the β-adrenergic receptor, adenylyl cyclase V and VI, and various membrane ion channels, regulate the reuptake and release of calcium from the sarcoplasmic reticulum, thereby regulating the systolic and diastolic capacity of the myocardium (3, 28, 29). The non-genomic effects are usually receptor independent and largely occur at the plasma membrane, regulating ion transporter activity, and are responsible, in part, for the ability of T3 to increase the heart rate (30). Although a hyperdynamic and hypermetabolic state induced by hyperthyroidism seems be more associated with chronic heart remodeling and heart failure, inflammatory infiltration, and network have been revealed as both the cause and the outcome of heart damage (31–33). Clinically, thyroid storms can induce acute heart failure and eventually lead to dilated cardiomyopathy, confirming that direct toxicity, and ion channel regulation by thyroxine accelerate myocardial inflammation (34, 35).

#### The Mechanism of Inflammation Dysfunction

The infiltration of inflammatory cells and the production of a large number of inflammatory factors aggravate the myocyte necrosis (8). Thus, we elaborate here on the underlying mechanism and characteristic of inflammation dysfunction in the heart induced by hyperthyroidism. Endomyocardial biopsy has documented that lymphocytic-dominated myocarditis is the commonest histological subtype of GD-related myocarditis, characterized by a dominant component of T lymphocytes and a variable number of macrophages (12, 22, 36). The lymphocytes are divided into several functional subtypes based on their surface markers: T lymphocyte [including CD4+ T effector cells, T helper cells (Th), regulatory T cells (Tregs), CD8+ T cell, B lymphocyte, and NK cells] (37). Infiltrated lymphocyte subpopulations contribute to the progression of myocarditis and subsequent cardiac remodeling. Using CD4 and CD8 knockout mice improves the prognosis and confirms their vital role in virus myocarditis (37). Furthermore, the transfer of cTnI-specific CD4+ effector T cells to healthy recipients causes severe inflammation, fibrosis, and cardiac dysfunction in animal models, suggesting an exclusive role for CD4+ T cells in myocarditis development and progression (38, 39). Th cells including Th1, Th2, and Th17 and FOXP3+ Treg cell may also contribute to this pathological process *via* complex regulatory networks (40). Song et al. found that a high frequency of Th2 cells and an increase of Th2 cytokines were characteristics of myocarditis patient hearts during the end stages of heart failure (41). The alteration of thyroxine influences the recruitment and activity of T lymphocytes (42). The enhanced expression of ICAM-1, VCAM-1, and the tissue inhibitors of metalloproteinases has been observed in GD patients, which promotes the infiltration of lymphocytes in the injured organs, including the heart (43). A hyperthyroid state leads to an increased activation of lymphocytes mediated by various factors including NF-κB, protein kinase C signaling pathways, and β-adrenergic receptor (44). However, the distribution and functional characteristics of these lymphocyte subsets in the occurrence and development of hyperthyroidism myocarditis are still not very clear and deserve further exploration in the future.

Additionally, attack by autoantibodies against the cardiomyocytes has been demonstrated to be the spark for the inflammatory disorders in autoimmune myocarditis (45). The discovery of heart reactive antibodies in the plasma or myocardium in autoimmune thyroiditis demonstrates that autoimmune-mediated tissue destruction in GD possibly contributes to autoimmune myocarditis (21). We have to ask how autoimmune antibodies against self-myosin are produced. In fact, cardiac myosin is well-concealed within the intracellular compartment. Its antigens, such as myosin heavy chain α (α-MHC), are expressed on thymic medullary epithelial cells as part of T cell selection under ischemia or toxic stimulation, which, in turn, launches an autoimmune sensitization (46, 47). A correlation between anti-myosin autoantibodies and deterioration of systolic and diastolic left ventricular function had been documented in patients with chronic myocarditis (48). Thus, more confirmed myocardial autoantibodies and their pathogenic mechanisms are worthy of identification in patients with GD.

Additionally, innate immune cells (macrophages, killer cells, dendritic cells, etc.) are also present in varying degrees in autoimmune myocarditis. Among them, monocyte–macrophage lineages are predominant in human and experimental myocarditis (49). Cardiac injury upon myocarditis results in an early recruitment of Ly6C^hi^ inflammatory monocytes from the circulation (50). Monocytes stimulated by Th1 cells tend to differentiate into pro-inflammatory M1 macrophages (51). Th2-associated cytokines, mainly IL-4 and−13, activate the anti-inflammatory M2 macrophage phenotype, which blunts the inflammatory response and promotes cardiac fibrotic healing (52, 53). Growing evidences have documented that excessive thyroxine enhances the proliferation and pro-inflammatory function of multiple cells in the innate immune system (44).

#### Conclusion

**Figure 4:**
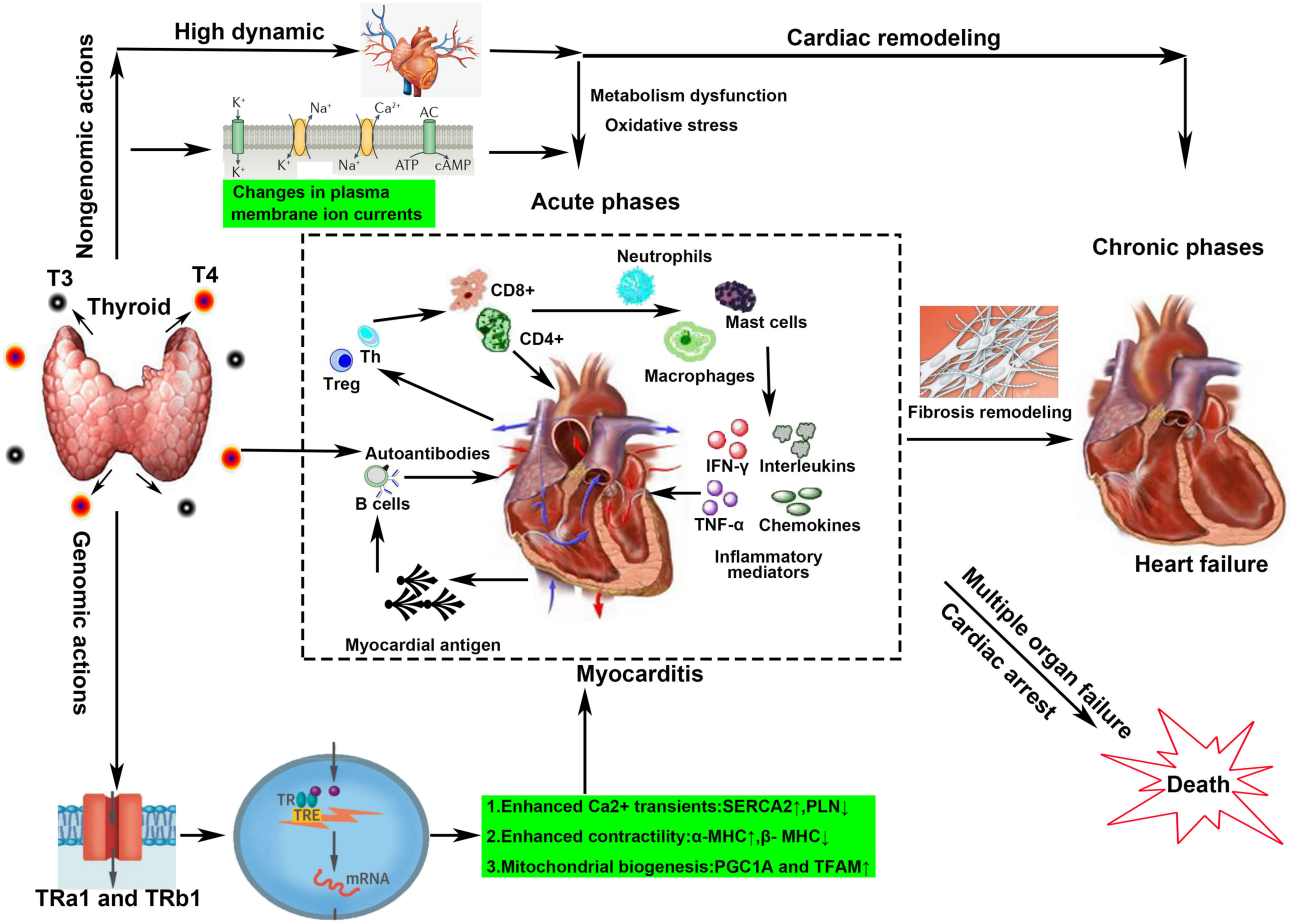
Overview of the possible mechanisms of hyperthyroidism-induced myocarditis. Both genomic and non-genomic actions contribute to the development of Graves' disease-induced myocarditis. Genomic actions mainly refer to binding to TH response elements in the promoter regions of TH-responsive genes. Non-genomic actions include high dynamic damage, ion channel alteration, and auto-attack from autoantibodies. All lead to a complex cascade of inflammation and sustained destruction of the heart muscle in the acute phases. In the chronic stage, fibrous repair, and structural remodeling are the main changes.

In conclusion, direct myocyte damage through hormonal effects or ion channel regulation and uncontrolled autoimmune response may be two coexisting mechanisms in the development of GD-induced myocarditis (Figure 4). However, their contributions vary at different stages of pathological development. In the acute phases, myocyte damage/necrosis is pronounced and promotes the production of inflammatory cascades and storms. In healing stages, myocyte damage becomes localized, and the release of inflammatory cytokines calms down (54). Lymphocytes give way to macrophages, and mesenchymal reparative tissue appears and is gradually substituted by replacement fibrosis (Figure 4).

### Animal Model

The establishment of animal models is of great significance for better understanding and elucidating the mechanisms behind Graves' disease-associated myocarditis. TH had been directly injected into different mice strains to investigate its association with cardiac structure and function. Short-term TH treatment (10 days) appeared to affect the heart rate primarily, with no change in heart size or function. However, moderate-length (2 months) and longstanding TH stimulation (10 months) in F1b hamsters resulted in significant cardiac hypertrophy and deleterious cardiac remodeling characterized by myocyte lengthening, chamber dilatation, decreased relative wall thickness, increased wall stress, and increased left ventricular (LV) interstitial fibrotic deposition (55, 56). However, whether myocarditis is involved in these processes has not been revealed in the above-mentioned study. In a hyperthyroid Wistar rat model induced by intraperitoneal injection with exogenous thyroxine for 4 weeks, cardiac lipid peroxides and serum endothelin-1 were increased, whereas cardiac superoxide dismutase, catalase, glutathione, and matrix metalloproteinase-2 were reduced in the hyperthyroid group. Unfortunately, myocardial inflammation (MPO level) and fibrosis (Masson trichrome staining) did not change significantly compared with the controls (57). Interestingly, hypothyroidism rats seem to be related with significant sterile inflammatory changes (leukocyte infiltration) in cardiac tissue (58). As mentioned above, the development of GD-associated myocarditis is associated not only with hyperthyroidism but also with the production of autoantibodies. A mouse model for GD was successfully established by three immunizations with recombinant adenovirus expressing the human TSHR, giving us hope to study the development of this myocarditis (59, 60). In a word, we have to admit that no animal model has been definitively considered suitable for the study of hyperthyroidism-associated myocarditis so far.

However, several murine models have been developed to simulate the development of autoimmune myocarditis (Table 3), which may help us to deeply track the mechanics of GD-associated myocarditis. Coxsackievirus B3 (CVB3), a non-enveloped single-strand positive-sense RNA virus, is often used to investigate viral myocarditis (71). Interestingly, heart-specific autoantibodies, especially anti-myosin IgG autoantibodies, are discovered in CVB3-infected mice (72, 73) and in patients with post-CVB3 myocarditis (74). Therefore, CVB3-infected mice were also used as a virus-induced autoimmune myocarditis model. Myosin and myosin peptides are also used to induce an autoimmune myocarditis model (Table 3). In 1987, Neu et al. described the immunization of mice with myosin-inducing myocarditis paralleled by high titers of myosin autoantibodies (63). Segments/smaller peptides from myosin also possess antigenicity and induce myocarditis. So far, at least seven different segments have been identified to induce autoimmune myocarditis (Table 3). Additionally, Göser et al. reported that immunization of mice with cardiac troponin I (cTnI) induced severe cardiac inflammation, fibrosis, and impaired LVEF (70).

**Table 3:** Overview of various autoimmune myocarditis models.

**Virus or peptide**	**Mouse strain**	**Brief methods** ** (dose/times/injection)**	**Days**	**References**
CVB3	A.CA/SnJ, A.SW/SnJ	0.1 ml of 10^5^ TCD_50_/once/intraperitoneally	15–21	(61)
EMCV	BALB/c	0.1 ml of 100 TCD_50_/once/intraperitoneally	14	(62)
Myosin	A/J, A.SW/SnJ, A.CA/SnJ	100 μg/twice*/subcutaneously	21	(63, 64)
α-Myosin	BALB/c	100 μg/twice/subcutaneously	21	(65)
Myosin fragment (1–1,032)	BALB/c	100 μg/twice/subcutaneously	56	(66)
Myosin fragment (1,074–1,646)	A/J	250 μg/once/subcutaneously	21	(67)
Myosin (334–352)	A/J	100 μg/twice/subcutaneously	21	(68)
Myosin (614–629)	BALB/CJ	100 μg/twice/subcutaneously	25–30	(69)
Myosin (614–643) Myosin (735–747) Myosin (947–960)	BALB/c	100 μg/twice/subcutaneously	21	(64)
Cardiac troponin I (cTnI)	A/J BALB/c	120 μg/twice/subcutaneously	21	(70)
cTnI (105–122) cTnI (131–148)	A/J	120 μg/twice/subcutaneously	28	(38)

EMCV, M variant of encephalomyocarditis virus; TCD_50_, 50% tissue culture infective dose; twice*, a week between the two injections.

### Diagnosis and Antidiastole

The proper diagnosis of hyperthyroidism-associated myocarditis is challenging due to a heterogeneous clinical presentation that ranges from no symptom, left ventricle systolic dysfunction, to mimicking the symptoms of acute myocardial infarction (AMI). Additionally, takotsubo myocardiopathy (TM), a rare transient abnormality of heart function with clinical manifestations like AMI but without a coronary artery disease, has been reported to be linked with hyperthyroidism, which also increases the difficulty of differential diagnosis (75, 76). Thus, AMI, takotsubo myocardiopathy, and myocarditis should be taken into consideration during the making of a differential diagnosis in this kind of patients.

Hyperthyroidism is prone to acute ischemic heart disease (77, 78). The proposed mechanisms responsible for coronary events in hyperthyroidism include a hypercoagulable and hypofibrinolytic state as well as hyperthyroidism-associated coronary vasospasm and increased myocardial oxygen demand (79, 80). Although many hyperthyroidism patients with typical chest pain, elevated levels of myocardial injury, and abnormal electrocardiograms have been diagnosed with myocardial infarction (MI) in the past, most of these patients are then shown to be free of coronary disorder as documented by both angiographic and postmortem studies (81–84). Thus, an important question haunts us as to whether these patients really suffered from myocardial infarction.

In order to comprehensively and carefully analyze the characteristics and incidence of patients in these reports, we reviewed and summarized the previous literatures in Supplementary Table 1. According to Takeshi's study, publications in English written before 1986 contained reports of 28 cases of concurrence of thyrotoxicosis and myocardial infarction (85). Seven of the patients showed normal coronary arteries, and the rest had been confirmed to have varying degrees of coronary disorders. In fact, only one patient was suspected of hyperthyroidism with myocarditis (86). She was a 22-year-old Navajo woman who experienced fatigue and dizziness after admission to a hospital. The ECG showed a progressive atrioventricular block, premature ventricular beat, and, eventually, cardiac arrest. Although she was suspected of hyperthyroidism-associated myocarditis, cardiac pathology was not performed. After 1986, we retrieved 43 cases of hyperthyroidism complicated with myocardial infarction in PubMed (Supplementary Table 1). Most of them (39/41) presented with active chest pain, palpitation, and other discomforts. Elevated markers of myocardial injury in circulation and myocardial infarction like ECG features, such as ST-segment elevation, depression, and pathological Q wave, linked them with MI. However, coronary angiography revealed that 23 patients had completely normal coronary arteries, seven patients presented with a reversible coronary artery spasm, and one patient had a myocardial bridge in the left anterior descending artery. One 25-year-old patient was suspected to be related to myocardial infarction because of old necrosis of the left ventricle and fresh ischemic necrosis of the ventricular septum after an autopsy. The other patients had different degrees of coronary atherosclerotic lesions, while only four patients had true occlusive atherosclerotic lesions. Obviously, most patients with normal coronary artery and myocardial injury are difficult to be explained by coronary ischemia. Coronary angiography is very important for ruling out AMI or coronary spasm. Unfortunately, our patient verbally rejected the recommendation for coronary angiography.

TM, also named as stress cardiomyopathy, transient left ventricular ballooning, or broken heart syndrome usually occurs following an emotional or physical stress. TM is more common in postmenopausal women with an average age of 66.8 years, accounting for as much as 89.8% of cases (87). Our patient is a young woman without any misfortune before being admitted to our hospital. Clinically, chest pain and dyspnea are the most common symptoms of TM. Moreover, the apical motility disorder shown in the echocardiogram and a ventriculogram with normal coronary arteries are the most common characteristics of TM. Moreover, transient akinesis, hypokinesis, or dyskinesis of ventricular myocardial segments in the echocardiogram, usually accompanied by a decrease of EF and an increase of BNP/NT-proBNP, is one of the major diagnostic criteria (88–90). However, the results of repeated echocardiographic examinations in July 21 and 23 are normal in our patient, and BNP is also in normal range. CMRI may be a useful tool to differentiate between TM and AMI but not suitable for TM and myocarditis. In TM, the MRI may show an isolated mid-wall or subepicardial pattern of LGE, which is similar to the MRI features of myocarditis but different to either subendocardial or transmural LGE observed in AMI. However, TM is an exclusive diagnosis, and the exclusion of ischemia, myocarditis, toxic damage, and tachycardia is one of the necessary criteria (88–90). In conclusion, our patient does not meet the diagnostic criteria for stress cardiomyopathy.

Myocarditis can also be manifested as a significant increase in myocardial injury markers and ischemia-like ECG changes (91). We only found five patients diagnosed as hyperthyroidism with myocarditis (Supplementary Table 1). One of them was diagnosed as acute myopericarditis. Two of these patients were diagnosed as having an extensive interstitial inflammation and necrosis of the myocardium at autopsy. The remaining two patients were diagnosed as having myocardial edema and delayed gadolinium enhancement by CMRI. Obviously, acute myocarditis complicating hyperthyroidism or thyrotoxicosis is underestimated. The main reason is that myocardial biopsy and CMRI are used less frequently in these patients with myocardial injury. Our patient is relatively young, without coronary heart disease risk factors and genetic predisposition. CMRI also indicates delayed gadolinium enhancement in T1 imaging and striped enhancement in T2 mapping. Thus, it is more likely to consider myocarditis with hyperthyroidism.

### Cardiac Biopsy or Cardiac Magnetic Resonance Imaging?

Myocardial biopsy is known to be the gold standard for the diagnosis of myocarditis, but it is rarely used in the clinic because of its invasive, serious complications, and false-negative results (92). CMRI can accurately display the pathological characteristics of acute myocarditis (AM) and has been widely used in the clinic (93, 94). Additionally, the non-radiation, non-invasive, high-resolution features, coupled with the emergence of various new technologies, make CMRI diagnosis more convenient and accurate (95). In 2009, the CMRI diagnostic criteria for AM, namely, LLC, was published (96, 97), which mainly targets the three core pathological features of AM: edema, congestion, and necrosis or fibrosis. The criteria include T2-weighted imaging, early gadolinium enhancement, and myocardial LGE. If more than two items are positive, AM can be diagnosed. The upgraded LLC in 2018 requires the following two requirements at the same time to diagnose AM: at least one sequence (T2-weighted imaging or T2 mapping) sensitive to edema and at least one T1 sequence (T1 mapping, extracellular volume, and myocardial delayed enhancement imaging) sensitive to necrosis (98, 99).

The differences between AM and MI on CMR are mainly as follows (100): (1) the T2-weighted images of AM show diffuse myocardial tissue edema that fails to match the distribution of coronary arteries, and it usually occurs in the epicardium or middle myocardium; MI is characterized by edema of the myocardial tissue in the area corresponding to the distribution of coronary arteries and is usually subendocardial or transmural; and (2) there is no abnormality in the resting myocardial perfusion of AM. The imaging of delayed myocardial enhancement shows multiple and scattered delayed enhancement of the epicardium or the middle myocardium, which is not consistent with the distribution of the coronary artery. It could exist alone or simultaneously in the interventricular septum and the anterior wall of the left ventricle, and the degree of enhancement gradually decreases with time. MI is characterized by subendocardial myocardial perfusion defect and delayed myocardial enhancement, consistent with the distribution of the coronary arteries, and the delayed enhancement usually does not go away.

However, CMRI also has its inevitable limitations. First of all, image quality can be limited by trigger problems (e.g., rhythm disturbances) and other artifacts (e.g., breath-holding and motion artifacts). In addition, the diagnostic sensitivity of borderline myocarditis is much lower than that of biopsy-proven active myocarditis, which may lead to omissions (44 vs. 84%) (101). They even recommended that only patients with myocardial necrosis of more than 2 g [corresponding CK levels: median, 229; range, 146–709 (U/L)] were eligible for CMRI (102). However, the correct detection rate of myocarditis was similar between the endomyocardial biopsy (EMB) (72/82, 88%) and CMRI (66/82, 80%; P $\frac{1}{4}$ 0.31) groups for all patients who were troponin-positive but without a coronary artery disease (103). Some researchers believe that the combination of CMR and EMB is probably the best option in improving diagnostic sensitivity because the combined use is more accurate than any of them alone (103, 104).

### Treatment

The clinical manifestations of myocarditis in patients are broad, ranging from the asymptomatic to minimal exertional dyspnea or palpitation, acute left heart failure, cardiogenic shock, and even sudden death (105). Therefore, the management of this condition must be personalized according to the severity in each case. The conventional treatment for hemodynamically stable patients emphasizes standard anti-heart failure regimens, including positive inotropic drugs, vasoactive drugs, beta-blockade, diuretics, ACEi/ARBs, and also aldosterone antagonists. It is also necessary to prevent and quickly respond to malignant arrhythmias such as malignant tachycardia and high atrioventricular block (106). Here we focus on the following three aspects on the treatment of hyperthyroidism-induced myocarditis: (1) restraint of thyrotoxicosis, (2) active response to cardiac damage, especially acute circulatory failure in fulminant myocarditis, and (3) immunosuppression or immunomodulatory therapy for excessive inflammatory storms.

#### Treatment of Thyrotoxicosis

Rapid blocking of thyrotoxicosis is the critical beginning to suppress the progress of hyperthyroidism-related myocarditis. Several mature drugs, including thioamides, iodine, β-blockers, and corticosteroids, are widely used to inhibit the synthesis and release of thyroid hormone, inhibit the peripheral effects of thyroid hormone, and increase thyroid hormone clearance (35). They can also alleviate heart injury and promote cardiac function by suppressing the thyrotoxic state and correcting the hemodynamic disturbances (75, 107). In one study, seven thyrotoxic patients with congestive heart failure showed an increase of mean LVEF from 28 to 55% after treatment for thyrotoxicosis (108). However, whether anti-thyroid drugs alone can be successful in the treatment of hyperthyroidism-induced myocarditis still needs more research.

#### Acute Supportive Treatment of Fulminant Myocarditis

Fulminant myocarditis (FM) is the most serious type of myocarditis, which is characterized by a rapid progressive decline in cardiac function and a high mortality rate. FM usually responds poorly to conventional vasoactive drug therapies as well as to standard heart failure, refractory heart failure, and cardiogenic shock treatments but relies on mechanical circulation support (MCS). The application of MCS devices, including intra-aortic balloon pump (IABP), peripheral venous–arterial extracorporeal membrane oxygenation (ECMO) or Impella, has resulted in better efficacy in FM patients from being <20 to 40–70% (106). Recently, the “life support-based comprehensive treatment regimen” proposed by our center has been verified to further reduce FM mortality from ~50 to <5% and shorten the hospitalization period to <2 weeks (106). The core idea of this treatment regimen is to strive for more recovery time for the exhausted heart by reducing or temporarily replacing its pumping function by using MCS devices. IABP is the most commercially available MCS device, can lower LV afterload, and can increase the tissue perfusion of important organs. However, due to the limited size of the balloon and less power of the pump, IABP can only provide about 15% of extra circulation support compared with the total circulation demand (109). ECMO is another useful MCS device which provides a more powerful circulation support or better oxygenation to venous blood and meets the basic demand of the body circulation. Impella is a small pump sent into the LV to drain blood and decrease the load. Although several reports had reported the benefit of Impella in treating cardiogenic shock, recent studies revealed that Impella might be associated with a higher risk of major bleeding and in-hospital mortality, accompanied by a decreased cost-effectiveness value (110, 111). Taken together, mechanical circulation support, especially IABP and ECMO, should be initiated if FM is the primary manifestation of hyperthyroidism-induced myocarditis.

#### Immunomodulatory Therapy

**Figure 5:**
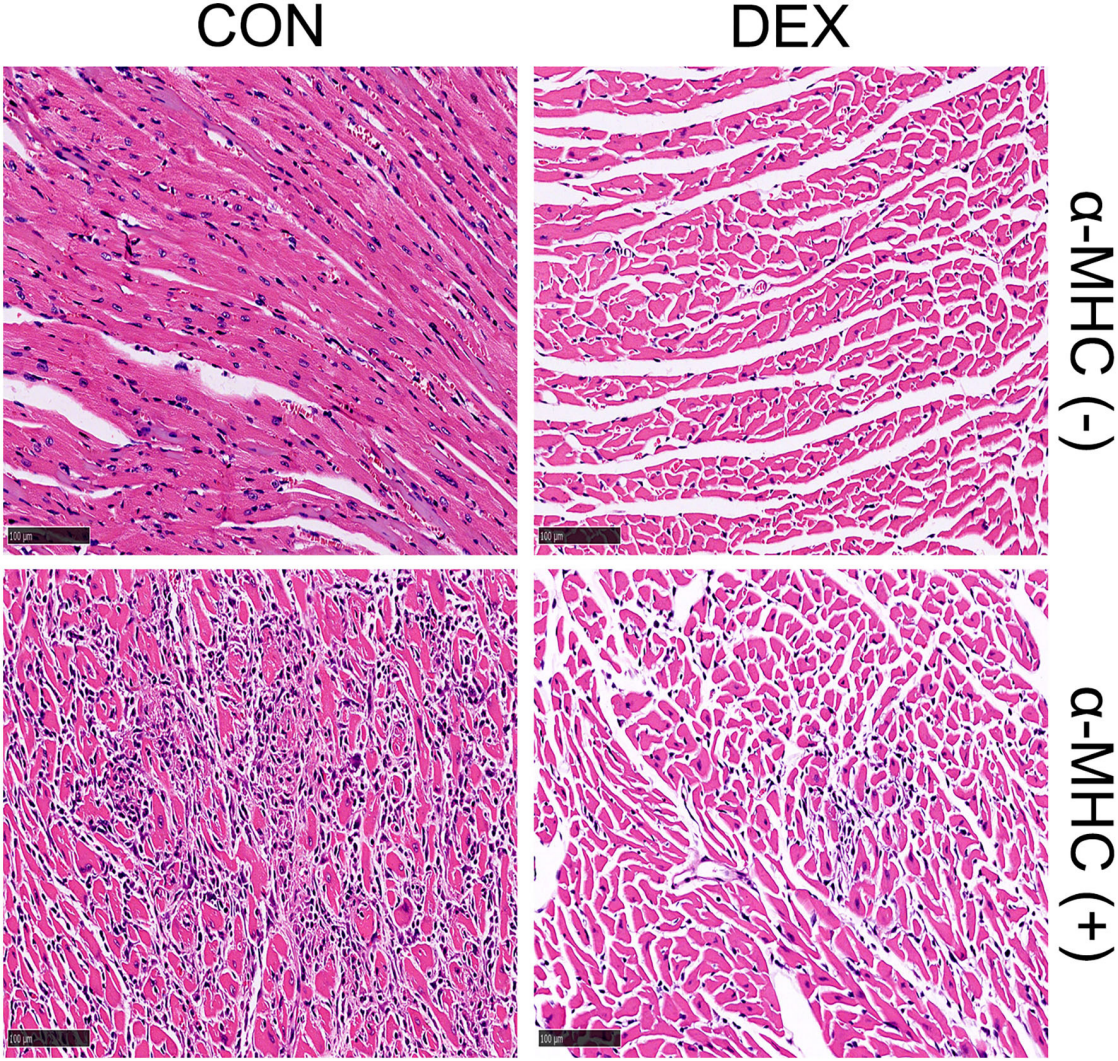
Representative heart sections of dexamethasone-treated autoimmune myocarditis induced by α-MHC immunization (α-MHC+). Establishment and treatment methods: in 0 and 7 days, respectively, 300 μg α-MHC (Ac-RSLKLMATLFSTYASADR-OH) was subcutaneously injected into BALB/C mice. Then, 0.75 mg/kg/day dexamethasone was given for intervention on the 16th to 20th day *via* intraperitoneal injection. The mice were sacrificed and their hearts were fixed with formalin for H&E staining on day 21.

With the consensus of the importance of inflammatory mechanisms or inflammatory storms in myocarditis (112), the prospect of immunosuppressive agents in the treatment of myocarditis has aroused the researchers' enthusiasm. Our clinical practice in virus-induced fulminant myocarditis suggested that “immunomodulatory therapy” may be more appropriate instead of “immunosuppressive therapy,” highlighting caution in the use of immunosuppressive agents such as azathioprine, cyclosporine, etc., but position on GC and immunoglobulin. In this study, we focus on the benefits of GC in myocarditis. For viral myocarditis, some people may worry that GC leads to viral replication at the first phase and aggravate the disease. This concern, though, seems to have been unfounded, as many large studies have shown that GC benefits from viral myocarditis with or without other immunosuppressants (113–115). Differently, the benefit of GC for autoimmune myocarditis is considered definite since it not only controls the severity of autoimmune diseases but also protects the heart through a pluripotent mechanism (65, 116, 117). A case–control study of 811 patients showed that early glucocorticoid treatment significantly reduced the in-hospital mortality of patients with thyroid storm (118). Additionally, we found that the early administration of dexamethasone can significantly reduce myocardial necrosis and inflammatory cell infiltration in α-MHC-induced autoimmune myocarditis animal model (Figure 5).

GC seems to be associated with cardio-protection and a range of possible favorable effects on inflammation regulation, heart metabolism, function, and survival (119). Firstly, GC translocates into the nucleus *via* a cytosolic glucorticoid receptor, binding to specific DNA binding sites (glucocorticoid-responsive elements), which promotes the production of anti-inflammatory and regulator proteins and inhibits the transcription of pro-inflammatory genes (120). Suppressing inflammation-related nuclear transcription factors such as activator protein 1, nuclear factor kB, interferon regulator factor 3, JAK, and STAT (121, 122) controls the expression of many inflammatory factors and adhesion molecules, such as interleukin (IL)-1, IL-2, IL-3, IL-6, IL-8, and IL-12, tumor necrosis factor-alpha, interferon-γ, granulocyte–macrophage colony-stimulating factor, MCP-1, VCAM-1, and ICAM-1 (123). Additionally, GC inhibits the activity of phospholipase A2 and cyclooxygenase, decreasing the production of inflammatory mediators, such as prostaglandins, leukotrienes, prostaglandin endoperoxides, and thromboxane (124). GC also causes programmed cell death in monocytes, macrophages, and T-lymphocytes via the upregulation of CD95 expression (125) but protects the myocardium from apoptosis by blocking pro-apoptotic signals (126). Moreover, GC's direct and rapid effects on cellular membranes (plasma and mitochondrial) result in the impairment of inflammatory and immune cell functions, such as phagocytosis, migration, antigen processing, and presentation via a non-genomic model (120, 127). On the other hand, GC also plays an important role on the control of thyrotoxicosis and regulation of T lymphocytes in GD. GC seems to decrease T4 secretion from the thyroid, although the efficacy and duration of this effect are unknown (128), and inhibit the peripheral conversion of T4 to T3 (129). Dexamethasone could effectively improve the function of Treg cells and sets up a new balance of T-helper1/T-helper2 in Graves' disease patients (130).

On the whole, except for direct relief of thyrotoxicosis, the pharmacological effects of corticosteroids include inhibition of immunological reactions, prevention of myocardial apoptosis, and reduction of cardiotoxicity by thyrotoxicosis and inflammatory cytokines. Our patient also proved that the use of GC can significantly reduce myocardial cell necrosis caused by hyperthyroidism.

## Author Contributions

LW collected and analyzed the data and wrote this manuscript. QL, WW, and NT helped to collect the data and treat this patient. DW designed the experiments and guided this study. NZ, YW, and DW checked this manuscript. All the authors have read and approved the manuscript.

## Conflict of Interest

The authors declare that the research was conducted in the absence of any commercial or financial relationships that could be construed as a potential conflict of interest.
