# Supplementary material for: Focus on Autoimmune Myocarditis in Graves' Disease: A Case-Based Review
Source: Front Cardiovasc Med. 2021 Jul 7;8:678645. doi: 10.3389/fcvm.2021.678645 (PMC8292634; doi:10.3389/fcvm.2021.678645)
Supplement: Supplementary file 1 [file Data_Sheet_1.docx]

Supplemental table1. From 1986 to 2021, all sporadic cases (excluding case series) of hyperthyroidism and myocardial infarction or myocarditis retrieved by PubMed were summarized.

| Patient ( age and sex) | Coronary risk factors | Heart-related symptoms | Auxiliary examination  (1.Troponin, 2.ECG, 3. CAG, 4.CMRI, 5. Autopsy ) | Diagnosis | Reference |
| --- | --- | --- | --- | --- | --- |
| 45-year-old woman | none | none | 2. a QS pattern in V4 and poor progression of R waves in leads V1-V3; 3.normal | MI complicating GD | 1 |
| 68-year-old woman | - | severe chest discomfort | 1.high; 2.marked T-wave inversion in leads I, II, aVL, and V2-V6; 3.normal | MI complicating thyrotoxicosis. | 2 |
| 52-year-old man | hypertension and CABG surgery | chest pain | 1.high; 2.ST segment elevation; 3. RCA, LAD and LCX were occluded and the grafts to LAD artery and to the obtuse marginal artery were 99% and 60% occluded. | MI complicating thyrotoxicosis | 3 |
| 50-year-old woman | - | none | 1.normal; 2.ST elevation in leads V2 and V3 and negative T waves in leads I, aVL, V3-V6; 3.normal. | MI complicating thyrotoxicosis | 4 |
| 48 year old woman | none | severe chest pain and dyspnoea | 1.high; 2.Q waves, and ST elevation in the anterior leads and T wave inversion in the lateral leads; 3. a significant Vasospastic narrowing of LM and 90% vasospastic stenosis in LAD. | Vasospastic MI complicating hyperthyroidism | 5 |
| 71-year-old woman | - | severe chest pain and dyspnoea | 1.high; 2.ST segment elevation in leads II, III, AVF, V5, and V6，ST segment depression in leads V2 to V4; 3.normal. | AMI complicating thyrotoxicosis | 5 |
| 42-year-old woman | none | chronic chest pain | 1.high; 2. anterior wall necrosis; 3.normal | MI complicating thyrotoxicosis | 6 |
| 28-year-old woman | smoking | sudden back pain and tachypnoea | 1.high; 2.nonspecific ST-segment changes with loss of R wave in the precordial derivations V1-3; 3.normal. | AMI complicating Thyrotoxicosis | 7 |
| 51-year-old woman | hypertension | severe retrosternal chest discomfort | 1.high; 2.diffuse ST-T abnormalities; 3.normal. | AMI complicating hyperthyroidism | 8 |
| 66-year-old woman | - | a prolonged episode of chest pain | 2.ST-segment elevations in leads V1 to V6; 3.total occlusion of the LAD artery and LCX artery due to coronary vasospasm. | Vasospastic MI complicating GD | 9 |
| 25-year-old woman | - | severe chest pain | 1.high; 2. ST-segment elevation in most leads; 3.A total proximal LAD occlusion. | AMI and cardiogenic shock complicating Thyrotoxicosis | 10 |
| 66-year-old woman | smoking | chest discomfort | 1.high; 2. disclosed acute myocardial ischeamia; 3. complete occlusion of LAD, and a critical obstruction of RCA. | AMI complicating GD | 11 |
| 27-year-old man | none | chest pain | 1.high; 2.ST segment elevation on II, III, avF and V5-V6; 3.normal. | AMI complicating Thyrotoxicosis | 12 |
| 40-year-old woman | none | Intermittent chest pain | 1.high; 2.T-wave inversions in the precordial leads 3. severe ostial vasospasm of the LM and RCA; 4.normal. | Vasospastic MI complicating GD | 13 |
| 28-year-old man | smoking | chest pain | 1.high; 2. ST segment elevation on II III avF; 3. a myocardial bridging of the LAD. | AMI complicating hyperthyroidism | 14 |
| 67-year-old woman | smoking | chest pain | 1.high; 2. T-wave inversion in V2–V6; 3.normal | AMI complicating subclinical hyperthyroidism | 15 |
| 78-year-old man | - | onset of chest pain and palpitations. | 1.high; 2.atrial fibrillation and STdepression in II avF v3-v6 leads 3.severe three vessels coronary artery disease. | AMI complicating subclinical hyperthyroidism | 16 |
| 51-year-old woman | - | multiple organ failure | 1.high; 2. elevated ST in leads II, III, aVF, and V2 - V5; 3.normal. | [MI and shock associated with thyrotoxicosis](https://www.geenmedical.com/article?id=21413419&type=true) | 17 |
| 63-year-old man | diabetes and smoking | palpitations | 1.high; 2. T wave inversion on v4-v5 leads; 3.normal | AMI complicating subclinical hyperthyroidism | 18 |
| 39-year-old woman | obesity | recurrent chest pain | 1.high; 2. wide range of ST-segment depression; 3. a massive spasm of LM and RCA | Vasospastic MI complicating GD | 19 |
| 35-year-old man | none | chest pain | 1.high; 2. ST-segment elevation in leads II, III, and aVF; 3.normal. | AMI complicating Thyrotoxicosis | 20 |
| 82-year-old woman | - | palpitation | 1.high; 2. ST-segment elevation in the inferolateral leads; 3. only minor nonobstructive disease in the LAD. | takotsubo cardiomyopathy complicating Thyrotoxicosis | 21 |
| 75-year-old woman | hypertension | chest pain and dyspnea | 1.high; 2. T wave inversion on II III avF and V1 - V6 leads; 3.normal. | AMI complicating subclinical hyperthyroidism | 22 |
| 48-year-old woman | none | crescendo angina | 1.High; 2.deep T inversion in leads V1 - V6; 3.normal. | non–ST-elevation MI complicating GD | 23 |
| 55-year-old woman | - | none | 1.high; 2. ST-segment elevation in leads II, III, aVF, as well as V1–V3; 3.normal. | AMI complicating Thyrotoxicosis | 24 |
| 25-year-old woman | none | cardiopulmonary arrest | 5. old necrosis on the anterior wall of the left ventricle  fresh ischemic heart attack on the interventricular septum | AMI complicating hyperthyroidism | 25 |
| 23-year-old woman | overweight | chest pain | 1.high; 2.Diffuse ST segment elevation; 3. distal LAD thrombosis with no sign of coronary dissection or atherosclerosis. | AMI complicating GD | 26 |
| 44-year-old woman | smoking and hypertension | mild dyspnea | 1.high; 2. ST segment elevation in the inferior leads; 3.normal | AMI complicating GD | 27 |
| 21-year-old man | none | chest pain and palpitation | 1.high; 2.ST-segment elevation in II, III, aVF; 3.normal. | AMI complicating hyperthyroidism | 28 |
| 66-year-old man | none | chest pain and palpitations | 1.high; 2.ST segment elevations in II, III, aVF, and V2 to V6; 3. normal | ST elevation MI complicating GD | 29 |
| 27-year-old woman | none | worsening chest discomfort | 1.high; 2.ST segment elevation in leads II, III and AVF; 3.severe RCA spasm. | Vasospastic MI complicating GD | 30 |
| 48-year-old woman | none | acute onset anterior chest pain | 1.high; 2.ST elevation over V1–3 with reciprocal ST depression over inferior leads (II, III, aVF)  3.severe vasospasm with total occlusion in the middle segment of both LAD and LCX. | Vasospastic MI complicating GD | 31 |
| 54-year-old male | none | severe left sided chest pain | 1.high; 2.ST-segment elevation in leads II, III aVF; 3.normal. | ST elevation MI complicating hyperthyroidism | 32 |
| 58-year-old man | none | severe and prolonged chest pain | 1.high; 2. ST-segment elevation in inferior leads; 3. a focal critical narrowing of RCA. | ST elevation MI complicating hyperthyroidism | 33 |
| 51-year-old woman | none | acute onset chest pain | 1.high; 2.normal; 3. 50% luminal stenosis in anomalous RCA. | Non-ST elevation MI complicating hyperthyroidism | 34 |
| 36-year-old woman | none | chest pain | 1.high; 2.ST-elevation; 3. distal blockages in the LAD and second obtuse marginal artery. | ST-elevation MI complicating GD | 35 |
| 37-year-old male | smoking | palpitation | 1.high; 2.Q waves and ST-segment elevations in II, III, aVF; 3.normal. | GD-associated silent MI | 36 |
| 31-year-old male | - | Severe chest pain and palpitations | 2.ST-elevation from V1-V4; 3.normal | ST elevation MI complicating hyperthyroidism | 37 |
| 26-year-old woman | none | none | 1.high; 2. ST elevation in leads II, III, and aVF; 3.normal | AMI complicating hyperthyroidism | 38 |
| 39-year-old man | none | intermittent chest pain | 1.high; 2. ST elevation in the anterolateral leads, new RBBB; 3. 40% LAD stenosis | ST-elevation MI complicating hyperthyroidism | 39 |
| 49-year-old woman | Smoking | chest pain | 1.high; 2. widespread ST-segment depression in the inferior leads and leads V4–V6 with inverted T waves and ST-segment elevation in lead aVR and V1; 3. severe RCA and LM coronary artery ostial vasospasm. | Vasospastic MI complicating GD | 40 |
|  |  |  |  |  |  |
| 37-year-old woman | - | acute cardiac arrest | 2. lowered ST-distances in V3-V6 and P-Q prolongation; 5 autopsy: diffuse interstitial inflammation. | an interstitial myocarditis complicating GD | 41 |
| 56- year -old man | diabetes | heart failure and cardiogenic shock. | 1.high; 2.ST segment elevation in I, II, V3–V6 and ST depression in aVR; 3. normal. | acute myopericarditis complicating GD | 42 |
| 46-year-old woman | - | heart failure | 1.high; 2. diffuse T-wave inversion; 5 autopsy:lymphocytic  Myocarditis. | Acute myocarditis complicating GD | 43 |
| 29‐year‐old man | none | acute onset chest pain and palpitations | 1.high; 2.ST‐segment convexity in V3‐V6 and marginal ST‐segment depression in III and aVF; 4.diffuse left ventricular  edema and subepicardial LEG. | Acute myocarditis complicating GD | 44 |
| 40-year-old man | none | acute chest pain | 1.high; 2.normal; 3.normal; 4. extensive subepicardial enhancement. | Acute myocarditis complicating GD | 45 |

Autopsy: The patients died quickly and a cardiac autopsy was performed; Coronary risk factors: mainly contain hypertension, diabetes, hyperlipidemia, obesity or smoking; CAG: coronary arteriography; RCA: right coronary artery; LAD: left anterior descending; LCX: [left circumflex artery](http://dict.youdao.com/w/left%20circumflex%20artery/#keyfrom=E2Ctranslation); LM: left main; CHD Coronary heart disease; CABG: coronary artery bypass graft; RBBB: right bundle branch block; LEG: Late gadolinium enhancement.

Reference

1. Nakano, T, Konishi, T, Futagami, Y, Takezawa, H. Myocardial infarction in graves' disease without coronary artery disease. *Japanese heart journal*. 1987;28:451-456

2. Bergeron, Ga, Goldsmith, R, Schiller, Nb. Myocardial infarction, severe reversible ischemia, and shock following excess thyroid administration in a woman with normal coronary arteries. *Archives of internal medicine*. 1988;148:1450-1453

3. Redahan, C, Karski, Jm. Thyrotoxicosis factitia in a post-aortocoronary bypass patient. *Canadian journal of anaesthesia = Journal canadien d'anesthesie*. 1994;41:969-972

4. Alexopoulos, D, Lazarou, N, Vagenakis, Ag. Electrocardiographic appearance of a non-q wave acute myocardial infarction in a patient with thyrotoxicosis. A case history. *Angiology*. 1995;46:353-356

5. Masani, Nd, Northridge, Db, Hall, Rj. Severe coronary vasospasm associated with hyperthyroidism causing myocardial infarction. *British heart journal*. 1995;74:700-701

6. Martínez Velasco, Mc, Lobo Palanco, J, Anguiano Baquero, P, Beunza Puyal, Mt. [acute myocardial infarct and thyrotoxicosis. A report of a new case]. *Revista espanola de cardiologia*. 1999;52:1019-1021

7. Timurkaynak, T, Aydogdu, G, Cengel, A. Acute myocardial infarction secondary to thyrotoxicosis. *Acta cardiologica*. 2002;57:439-442

8. Gowda, Rm, et al. Acute myocardial infarction with normal coronary arteries associated with iatrogenic hyperthyroidism. *International journal of cardiology*. 2003;90:327-329

9. Lassnig, E, Berent, R, Auer, J, Eber, B. Cardiogenic shock due to myocardial infarction caused by coronary vasospasm associated with hyperthyroidism. *International journal of cardiology*. 2003;90:333-335

10. Opdahl, H, Eritsland, J, Søvik, E. Acute myocardial infarction and thyrotoxic storm--a difficult and dangerous combination. *Acta anaesthesiologica Scandinavica*. 2005;49:707-711

11. Owecki, M, Sowiński, J. Acute myocardial infarction during high-dose methylprednisolone therapy for graves' ophthalmopathy. *Pharmacy world & science : PWS*. 2006;28:73-75

12. Grabczewska, Z, Białoszyński, T, Kubica, J. [acute myocardial infarction in a patient with iatrogenic thyrotoxicosis--a case report]. *Kardiologia polska*. 2007;65:280-282

13. Patel, R, et al. Hyperthyroidism-associated coronary vasospasm with myocardial infarction and subsequent euthyroid angina. *Thyroid : official journal of the American Thyroid Association*. 2008;18:273-276

14. Patanè, S, et al. Acute myocardial infarction in a young patient with myocardial bridge and elevated levels of free triiodothyronine. *International journal of cardiology*. 2009;132:140-142

15. Patanè, S, Marte, F, Di Bella, G, Turiano, G. Acute myocardial infarction and subclinical hyperthyroidism without significant coronary stenoses. *International journal of cardiology*. 2009;134:e135-137

16. Patanè, S, Marte, F. Paroxysmal atrial fibrillation during acute myocardial infarction associated with subclinical hyperthyroidism, severe three vessels coronary artery disease and elevation of prostate-specific antigen after turp. *International journal of cardiology*. 2010;138:e28-30

17. Iwańczuk, W. [myocardial infarction and shock associated with thyrotoxicosis]. *Anestezjologia intensywna terapia*. 2010;42:142-146

18. Patanè, S, Marte, F. Atrial fibrillation and acute myocardial infarction without significant coronary stenoses associated with subclinical hyperthyroidism and erythrocytosis. *International journal of cardiology*. 2010;145:e36-39

19. Kuang, Xh, Zhang, Sy. Hyperthyroidism-associated coronary spasm: A case of non-st segment elevation myocardial infarction with thyrotoxicosis. *Journal of geriatric cardiology : JGC*. 2011;8:258-259

20. Kim, Hj, et al. Thyrotoxicosis-induced acute myocardial infarction due to painless thyroiditis. *Thyroid : official journal of the American Thyroid Association*. 2011;21:1149-1151

21. Zuhdi, As, et al. Takotsubo cardiomyopathy in association with hyperthyroidism. *Medicina (Kaunas, Lithuania)*. 2011;47:219-221

22. Patanè, S, Marte, F, Sturiale, M. Acute myocardial infarction without significant coronary stenoses associated with endogenous subclinical hyperthyroidism. *International journal of cardiology*. 2012;156:e1-3

23. Lee, Cp, et al. Recurrent vasodilator-refractory acute coronary syndrome as the exclusive manifestation of graves disease. *The American journal of emergency medicine*. 2012;30:1656.e1655-1659

24. Kauffels, A, Lee, Io, Schilling, Mk, Slotta, Je. Thyrotoxicosis after parathyroidectomy mimicking myocardial infarction: A case report and review of the literature. *Clinical research in cardiology : official journal of the German Cardiac Society*. 2012;101:687-690

25. Hama, M, et al. A case of myocardial infarction in a young female with subclinical hyperthyroidism. *International journal of cardiology*. 2012;158:e23-25

26. Bouabdallaoui, N, Mouquet, F, Ennezat, Pv. Acute myocardial infarction with normal coronary arteries associated with subclinical graves disease. *The American journal of emergency medicine*. 2013;31:1721.e1721-1722

27. Beedupalli, J, Akkus, Ni. Concealed pheochromocytoma presenting as recurrent acute coronary syndrome with stemi : Case report of a patient with hyperthyroidism. *Herz*. 2014;39:476-480

28. Zheng, W, et al. Painless thyroiditis-induced acute myocardial infarction with normal coronary arteries. *The American journal of emergency medicine*. 2015;33:983.e985-910

29. Zhou, D, et al. Severe hyperthyroidism presenting with acute st segment elevation myocardial infarction. *Case reports in cardiology*. 2015;2015:901214

30. Nannaka, Vb, Lvovsky, D. A rare case of gestational thyrotoxicosis as a cause of acute myocardial infarction. *Endocrinology, diabetes & metabolism case reports*. 2016;2016

31. Chang, Kh, et al. Vasospastic myocardial infarction complicated with ventricular tachycardia in a patient with hyperthyroidism. *International journal of cardiology*. 2017;234:143-145

32. Rymer De Marchena, I, et al. Thyrotoxicosis mimicking st elevation myocardial infarction. *Cureus*. 2017;9:e1323

33. Menichetti, F, et al. St-segment elevation acute myocardial infarction associated with hyperthyroidism: Beware of coronary spasm! *Journal of cardiovascular medicine (Hagerstown, Md.)*. 2017;18:798-799

34. Zeitjian, V, et al. Manifestation of non-st elevation myocardial infarction due to hyperthyroidism in an anomalous right coronary artery. *International journal of general medicine*. 2017;10:409-413

35. Kowtoniuk, R, Eldredge, N, Puntagunta, R. Alpha-2 antiplasmin-associated aortic valve thrombus presenting as a stemi in a patient with graves disease. *BMJ case reports*. 2018;2018

36. Li, C, et al. A silent myocardial infarction with normal coronary arteries associated with graves' disease. *Heart & lung : the journal of critical care*. 2019;48:347-350

37. Krishnan, Gd, Yahaya, N, Yahya, M. Hyperthyroidism presenting as st elevation myocardial infarction with normal coronaries - a case report. *Journal of the ASEAN Federation of Endocrine Societies*. 2019;34:92-94

38. Wang, L, Yang, J, Zheng, J, Gu, X. Acute myocardial infarction in pregnancy: Spasm caused by hyperthyroidism? *The Journal of international medical research*. 2019;47:2269-2273

39. Mubasher, M, Patel, A, Magdi, M, Hamid, T. Stemi after dobutamine stress echocardiography in hyperthyroid state. *Case reports in cardiology*. 2019;2019:7434071

40. Klomp, M, Siegelaar, Se, Van De Hoef, Tp, Beijk, Mam. A case report of myocardial infarction with non-obstructive coronary artery disease: Graves' disease-induced coronary artery vasospasm. *European heart journal. Case reports*. 2020;4:1-5

41. Ortmann, C, Pfeiffer, H, Du Chesne, A, Brinkmann, B. Inflammation of the cardiac conduction system in a case of hyperthyroidism. *International journal of legal medicine*. 1999;112:271-274

42. Kukla, P, et al. [myopericarditis complicated with cardiogenic shock mimicking acute coronary syndrome with st elevation in a patient with hyperthyroidism and diabetes mellitus]. *Kardiologia polska*. 2008;66:982-986; discussion 986

43. Chen, Yt, Yang, Gg, Hsu, Yh. Thyroid storm and lymphocytic myocarditis. *Internal medicine (Tokyo, Japan)*. 2010;49:593-596

44. Lancaster, St, et al. Acute autoimmune myocarditis as a manifestation of graves' disease: A case report and review of the literature. *Clinical case reports*. 2019;7:1489-1493

45. Demoulin, R, et al. [acute autoimmune myocarditis secondary to graves' disease: A case report]. *La Revue de medecine interne*. 2020;41:206-209
